# Effectiveness of ATP bioluminescence assay for presumptive identification of microorganisms in hospital water sources

**DOI:** 10.1186/s12879-017-2562-y

**Published:** 2017-06-30

**Authors:** Máira Gazzola Arroyo, Adriano Menis Ferreira, Oleci Pereira Frota, Marcelo Alessandro Rigotti, Denise de Andrade, Natalia Seron Brizzotti, Jacqueline Tanury Macruz Peresi, Elza Maria Castilho, Margarete Teresa Gottardo de Almeida

**Affiliations:** 1 Graduate Program in Microbiology, São Paulo State University, Street Cristóvão Colombo, 2265, 15054-000, São José do Rio Preto, São Paulo, Brazil; 2 Master and Doctoral Graduate Program in Health and Development in the West Central Region and Master Graduate Program in Nursing, Federal University of Mato Grosso do Sul, Av. Costa and Silva, s/n, 79070-900, Campo Grande, Mato Grosso do Sul, Brazil; 3 Undergraduate Program in Nursing, Federal University of Mato Grosso do Sul, Av. Ranulpho Marques Leal, 3484, 79610-100, Três Lagoas, Mato Grosso do Sul, Brazil; 4 Department of General and Specialized Nursing, Ribeirão Preto College of Nursing, WHO Collaborating Centre for Nursing Research Development, University of São Paulo, Av. Bandeirantes, 3900, 14040-902, Ribeirão Preto, São Paulo, Brazil; 5 Department of Infectious and Parasitic Diseases, Faculty of Medicine of São José do Rio Preto, Av. Brg. Faria Lima, 5416, 15090-000, São José do Rio Preto, São Paulo, Brazil; 6 Center of Regional Laboratory of São José do Rio Preto, Adolfo Lutz Institute, Street Rua Alberto Sufredini Bertoni, 2325, 15060-020, São José do Rio Preto, São Paulo, Brazil

**Keywords:** Adenosine triphosphate, Bacteria, Fungi, Hospital, Water

## Abstract

**Background:**

Laboratory analysis of organisms in water include arduous methods, such as the multiple tube and membrane filter. The ATP bioluminescence system, proposes a new way of measuring cellular material in water by measuring adenosine triphosphate (ATP) levels, which are expressed in relative light units (RLU). The ATP bioluminescence assay has been increasingly used to assess the microbiological safety of the hospital environment. However, there are few studies investigating the use of this methodology to evaluate the microbiological quality of water. The objective of the present study was to verify whether ATP, as measured by the 3 M*™* Clean-Trace Water™ ATP test, can be used as an alternative tool for presumptive testing for the presence of microorganisms in hospital water.

**Methods:**

Water samples (*N* = 88) were collected from faucets (74) and water purifiers (14) in a university hospital. The sample were filtered by the membrane filter technique (100 mL for bacterial analysis and 100 mL for fungal analysis) and then submitted to ATP bioluminescence assay to the determine quantity of RLU in each sample. In order to compare RLU and the presence of microorganisms, a receiver operating characteristic (ROC) curve was used to calculate sensitivity and specificity (levels higher than 90% were considered significant). In addition, control tests were conducted to compare RLU to the quantities of bacterial and fungal organisms added to distilled water (ANOVA and Tukey’s tests; *p* ≤ 0.05). This inoculum was compared to RLU emission, and the data were analyzed by calculating the Pearson’s correlation coefficient, with a 95% confidence interval.

**Results:**

In the present study, 94.3% of the water samples presented bacterial growth. Of these, 15.6% showed heterotrophic bacteria above recommended levels and fungal contamination was detected in 55.6% of samples. Sensitivity and specificity of the samples were not significant (< 90%), and the correlation between ATP and the presence of these microorganisms in the samples (hospital water) was not significant, whereas, in distilled water, the results revealed a significant difference (*p* < 0.0001).

**Conclusions:**

These results demonstrated that the ATP test cannot be used as an alternative tool for presumptive assessment of the presence of microorganisms in water.

## Background

The presence of organisms in drinking water is assessed in laboratory analysis by qualitative and quantitative criteria [1]. Initial investigations have included consistent but arduous methods, such as the multiple tube method, involving serial analysis of water samples. Subsequent studies have included alternative, less laborious methods of quantitative and qualitative analysis that use chromogenic and fluorogenic substrates and maintain high specificity and sensitivity along with speed [2, 3]. Among these methods, the membrane filter method stands out. It is considered a feasible laboratory procedure that allows for bacterial colony counts [1].

Another methodology, similar to the ATP bioluminescence system, has been proposed as a new method of measuring cellular material in water by measuring levels of adenosine triphosphate (ATP) [4].

One of the major advantages of ATP detection is that it is fast and easy to carry out [5–7]. However, there are also some disadvantages, such as low sensitivity and inability to differentiate extracellular and intracellular ATP [7–10]. Dead cells, blood, excretions, human secretions, food and other organic materials are considered extracellular ATP, whereas intracellular ATP is detected by means of viable microorganisms [7, 10, 11].

Studies comparing the traditional methodology (colony-forming units, − CFU) with ATP detection have shown divergent results, indicating the need for more research to establish better understanding of the relationship between microorganism counts and ATP. Lee et al. [12] and Bushon et al. [13] found good correlations between ATP and CFU, but other studies [14, 15] found no significant association.

Considering the high incidence of waterborne diseases in the general population, it is necessary to establish faster and more specific protocols for microbiological investigation of water, in order to adopt control measures. The objective of the present study was to verify whether ATP bioluminescence can be used as an alternative methodology for presumptive testing for presence of microorganisms in water, by confirming that there is a correlation between RLU and the presence of these microorganisms.

## Methods

### Collection of samples

Water samples of 200 mL each (*n* = 88) were collected from faucets (74) and water purifiers (14) (both of subterranean origin) at a university hospital in the state of São Paulo, Brazil; the origin of the water within the hospital is shown in Table 1. For each sample, 100 mL was submitted to bacterial analysis and 100 mL to fungal analysis.

**Table 1:** Distribution of the results according to the origin of the samples

Location	Number of samples		Number of samples with fungi		Number of samples with bacteria >500 CFU/mL		Number of samples with free chlorine residual within the limits	
Ground floor:	F	P	F	P	F	P	F	P
Hemodialysis	7	1	2	0	0	0	7	1
1st floor: Surgical center	6	2	4	1	0	2	6	0
2nd floor: Department of infectious diseases	6	2	4	2	2	2	4	0
3th floor: Oncology	13	3	6	2	1	1	13	0
4th floor: Pediatrics	7	1	6	0	0	0	7	0
5th floor: Cardiology	6	2	3	0	0	2	6	0
6th floor: Post-operative room	6	2	5	0	0	2	6	0
7th floor: Intensive care	15	1	7	0	0	1	14	0
8th floor: Transplant	8	0	7	0	0	0	8	0
Total	74	14	44	5	3	10	71	1

*F* faucets, *P* purifiers, *CFU* colony-forming units

Eight samples were collected from each floor (ground to 8th floors), with the exception of the 3rd and 7th floors, where 16 samples were collected from each. The samples were placed in sterile Whirl-Pak® Thio-Bags® with sodium thiosulfate 1.8% to neutralize the disinfectant activity of chlorine in chlorinated samples. The bags were transported in isothermal boxes to the hospital’s microbiology laboratory [1, 16].

### Filtration of the samples and microbiological analysis

According to standard methodology [1, 16], immediately after sample collection, 100 mL of each sample were filtered through a nitrocellulose membrane (0.45 μm) plated in plate count agar medium (Difco Laboratories), and incubated for 48 h at 35 °C.

At the same time, the other 100 mL of each sample were filtered. After the filtration process, the membrane was transferred to a petri dish with Sabouraud dextrose agar (Difco Laboratories) and incubated for 24 h at 30 °C [17, 18]. After this period, the membrane was removed from the original dish and maintained under the same thermal incubation conditions for 15 days. For purposes of comparison with the standard methods, the results were divided by 100 to express CFU/mL.

### Physical analysis

Free chlorine residual content was recorded immediately after sample collection. To determine this content, 10 mL of the sample and 0.5 mL of the reagent NN diethyl-p-phenylenediamine were added to a cuvette. The mixture was stirred for homogenization and measured with an electronic colorimeter device (HI96711C - Hanna Instruments). To conform to the standards required by Regulation 2.914/11, samples must have minimum concentrations of free chlorine residual of 0.2 mg/L and maximum limits of 2 mg/L [1].

### ATP (bioluminescence) test and statistical analysis

Immediately after sample collection, ATP bioluminescence (3 M*™* Clean-Trace Water™ ATP system) was used to the quantify RLU in all samples. In this system, a specialized rod is inserted into the water sample and then placed in a tube with a reagent that unleashes a bioluminescence reaction; luciferase is the revealing enzyme and luciferin, the substrate. Light is emitted in the presence of ATP, which is detected by specialized equipment (a luminometer). Concentrations of ATP are directly proportional to the quantity of cellular material and are expressed in relative light units (RLU) [4].

In order to compare RLU and the presence of microorganisms, a receiver operating characteristic (ROC) curve was used to calculate sensitivity and specificity: levels higher than 90% were considered significant.

Control tests were conducted in order to compare RLU with the quantity of bacterial and fungal organisms present in distilled water, which is free of microorganisms and cellular material (ANOVA and Tukey’s tests, when *p* was significant: *p* ≤ 0.05). Thus, pure *Staphylococcus aureus* (*American Type Culture Collection* - ATCC 25923) and *Candida parapsilosis* cultures (ATCC 22019) were reactivated on nutrient agar for 24 h at 37 °C. Colonies were placed in tubes containing 5 ml sterile distilled water to achieve concentrations (0.5 to 10) according to the McFarland standards. Dilutions were performed to achieve a dilution of 10^−10^ from the lower concentration. From these concentrations, 100 μl were transferred to polystyrene dishes and the absorbance value was checked (Biospectro spectrophotometer). This inoculum was compared with RLU emission, and the data were analyzed by calculating Pearson’s correlation coefficient, with a 95% confidence interval.

## Results

Table 1 shows the distribution of the results obtained, according to the origin of the samples. Eighty-three (94.3%) samples presented bacterial growth: 69 (83.1%) from faucets and 14 (16.9%) from purifiers. Of these, 13 (15.6%) presented heterotrophic organism levels above those recommended by the Brazilian Ministry of Health (≤500 CFU/mL). There was also fungal contamination in 49 (55.6%) water samples.

**Fig. 1:**
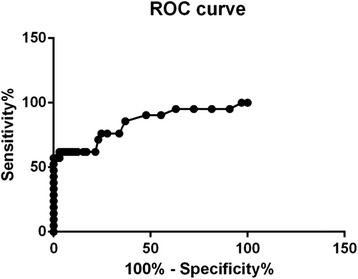
ROC curve: RLU sensitivity versus specificity

In relation to the data for RLU, 36 samples presented exclusive bacterial growth. This can be explained by the fact that no fungal growth was observed in the Sabouraud agar medium plates, which favor fungal growth. If neither yeast nor mold grew in the plates with a medium that is selective for fungi, it can be concluded that there was only bacterial growth in the plate count agar medium, with a mean RLU value of 14.4. Only two samples presented exclusive fungal growth, with a mean RLU of 23. Water samples that presented simultaneous growth of two types of microorganisms (*N* = 47) had a mean RLU value of 16.5. For the samples that showed no microorganism growth (*N* = 3), the mean RLU value was 8.6. The minimum and maximum RLU values found in the samples were 4 (3rd and 7th floor) and 141 (2nd floor). An ROC curve was used to establish a cutoff point of RLU values to detect contaminated water. However, the sensitivity and specificity of the samples were not significant, as most had results lower than 90% (Fig. 1).

**Fig. 2:**
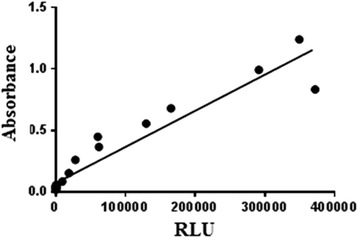
Correlation between microbial concentration of *S. aureus* (absorbance) and RLU

**Fig. 3:**
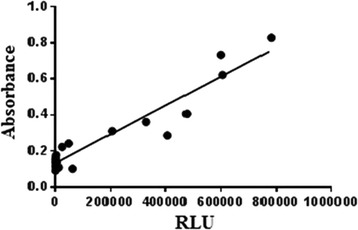
Correlation between microbial concentration of *Candida parapsilosis* absorbance) and RLU

Pearson’s correlation coefficient between RLU measurement and microbial concentration (absorbance) in distilled water revealed results with significant differences, *p* < 0.0001 (Figs. 2 and 3).

Minimum and maximum limits of detection are 10^−10^ and 10. For *S. aureus,* the corresponding RLU value for the 10^−10^ dilution was 18; for the concentration of 10, it was 349,333*.* For *C. parapsilosis*, the corresponding RLU values were 37 and 780,198 for the minimum and maximum limits of detection, respectively.

## Discussion

Lack of significant correlations between RLU and the presence of bacteria and fungi (Fig. 1) could be due to several factors, such as variations in microbial cell size and cell development stages, as has been described by other authors [7]. A sample with few yeast cells can result in high levels of RLU (ATP), and one with many yeast cells can present low levels of RLU. The effect of environmental stress on RLU emission has been investigated in other studies [19, 20]. On study examined the effects of hydric stress and nutritional conditions on *Pseudomonas paucimobilis* present in soil. The findings demonstrated that ATP content increased in dry soil or with the introduction of glucose and ammonium salts [19].

Furthermore, in the present study, RLU was detected even in samples in which fungi and bacteria were not isolated. This may be explained by the presence of other microorganisms, such as protozoa or anaerobic bacteria. In addition, the chlorine present in water (Table 1) could inhibit some microorganisms for culture, but ATP was present. Similarly, some bacteria that would grow at lower or higher temperatures did not grow in the culture media, but ATP was found.

This lack of correlation could also have occurred because the system does not make a distinction between intracellular and extracellular ATP. Both intracellular and extracellular ATP were measured, which does not correspond to the microorganisms present in the samples. Biological systems have other nonbacterial sources of ATP and, consequently, microbial ATP represents only a portion of the ATP responsible for bioluminescence. Interference from ATP of nonmicrobial origin may limit the sensitivity of the method and lead to false positives [21–23]. However, whether the samples included intracellular or extracellular ATP, the results of the present study indicate that, in addition to including viable microorganisms, the samples also contained secretions and excretions, making the water unfit for consumption and implying health problems.

In relation to the average RLU in the samples with exclusive fungal growth, there was higher-than-average RLU in samples with exclusive bacterial growth, or growth of both bacteria and fungi. A possible explanation is that in the samples with exclusive fungal growth, free chlorine residual content was within the established limits (Table 1), and some bacteria that were present could not be grown, but their ATP was detected, indicating high levels of RLU.

Perhaps for these reasons, discrepancies between the results of the ATP bioluminescence and the presence of microorganism in the samples indicate that this correlation is still questionable and needs more research in order to gain consensus.

When compared to samples of distilled water (control test) contaminated with ATCC from *S. aureus* and *C. parapsilosis,* and considering the correspondence of these samples’ absorbance with RLU, the results were significant; when the inoculums increased, so did RLU levels (Figs. 2 and 3). In this situation, distilled water does not have interferents or microorganisms other than those that have been introduced, which could explain the significant results obtained.

## Conclusions

The ATP bioluminescence system used in this study showed low correlations with microbiological cultures. Therefore, this technology cannot be used as an alternative methodology for presumptive testing for the presence of microorganisms in water.

## Abbreviations

ATCC: American Type Culture Collection
ATP: Adenosine triphosphate
*C. parapsilosis*: *Candida parapsilosis*
CFU: Colony-forming units
DPD: NN diethyl paraphenylene diamine
mL: Milliliter
RLU: Relative light units
ROC: Receiver operating characteristic
*S. aureus*: *Staphylococcus aureus*
